# Population-Based Health Survey in the municipality of São Paulo 2024: methodological and operational aspects

**DOI:** 10.1590/1980-549720260022

**Published:** 2026-06-15

**Authors:** Chester Luiz Galvão Cesar, Katia Cristina Bassichetto, Zilda Pereira da Silva, Marilia Cristina Prado Louvison, Regina Mara Fisberg, Maria Cecilia Goi Porto Alves, Maria Mercedes Loureiro Escuder, Gizelton Pereira Alencar, Regina Tomie Ivata Bernal, Edige Felipe de Sousa Santos, Olinda do Carmo Luiz, Moises Goldbaum

**Affiliations:** I Universidade de São Paulo, School of Public Health – São Paulo (SP), Brazil.; II Secretaria de Estado da Saúde de São Paulo, Instituto da Saúde – São Paulo (SP), Brazil.; III Universidade Federal de Minas Gerais, School of Nursing, Graduate Program – Belo Horizonte (MG), Brazil.; IV Universidade de São Paulo, School of Medicine – São Paulo (SP), Brazil.

**Keywords:** Health surveys, Methods, Cross-sectional studies, Chronic diseases, Health services

## Abstract

This paper presents the methodological procedures adopted for the construction and development of the population-based household health survey (ISA Capital-SP 2024), including its planning, sampling, and operational aspects for field feasibility. The data were collected using stratified probability sampling, with two-stage cluster sampling: at the census tract and household level. The strata were formed by the six health regions of the municipality of São Paulo (MSP). Individuals aged 10 or older living in permanent private households in the MSP's urban area were interviewed. The questionnaire consisted of 13 thematic blocks, including: reported morbidity, service use, preventive exams, immunization, medication use, and health behaviors. Health surveys contribute to studies of health trends and the identification of different patterns of health service use by the population, complementing data from official information systems. The ISA constitutes an important instrument for monitoring health conditions, planning, and formulating public policies at the municipal and intra-municipal levels, especially given the need to generate regional indicators for a city with the social and geographic inequalities of the MSP.

## INTRODUCTION

The production of information from diverse sources enables the expansion of knowledge regarding the complexity of health problems, allowing the generation of indicators for continuous monitoring. Health surveys broaden the analytical scope of data derived from information systems and enable the measurement of behaviors that influence health habits and risk exposures, the assessment of patterns of healthcare utilization, the quantification of health conditions associated with a high burden of morbidity, and the stratification of indicators across population subgroups to highlight health inequalities^1,2^.

In this context, population-based health surveys have held a prominent position in the generation of health information since the 1960s across several European countries, the United States, and China, encompassing diverse settings and involving hundreds of thousands of households and participants^3–9^.

In Brazil, there is an established policy that recognizes the need for periodic surveys to generate information not available from other sources^10,11^. Since the 1970s, several household surveys, including health surveys, have been conducted. The main initiatives include the Family Budget Surveys (*Pesquisa de Orçamentos Familiares* – POF); the National Demographic and Health Survey (*Pesquisa Nacional de Demografia e Saúde* – PNDS); the Health Supplements of the National Household Sample Survey (*Pesquisa Nacional por Amostra de Domicílios* – Pnad); the National Health Survey (*Pesquisa Nacional de Saúde* – PNS), which provides data on health status and lifestyles, as well as on access to and use of services, preventive actions, continuity of care, and healthcare financing; the National Oral Health Survey (*Pesquisa Nacional de Saúde Bucal –* SB Brasil); and the National School Health Survey (*Pesquisa Nacional de Saúde do Escolar* – PeNSE), an important initiative for understanding adolescent health and well-being^12–16^.

Particularly in the health sector, given the country's continental dimensions, surveys have enabled the expansion of knowledge on variations in national and regional health indicators, as well as a deeper examination of issues requiring more specific investigation. The information generated on determinants, conditioning factors, and health needs supports the formulation and improvement of policies within the Brazilian Unified Health System (*Sistema Único de Saúde* – SUS)^14^.

The state of São Paulo also has a longstanding tradition of conducting health surveys, beginning in Ribeirão Preto in 1974 with the Continuous Survey of Health Conditions through Household Interviews (the first survey in the health field in Brazil^17^). This was followed by the Morbidity Project in 1990, conducted in the southwest region of Greater São Paulo^18,19^, and the Multicenter Survey of the State of São Paulo in 2002, carried out in Botucatu, Campinas, Greater São Paulo, and in a health district of São Paulo^20^. Additional municipal experiences have also been developed, such as the Campinas Health Survey and the ISA Capital-SP (2003, 2008, and 2015) conducted in the municipality of São Paulo (MSP). A notable feature of the latter is its capacity to address analytical needs at the intramunicipal management level. Since 2015, ISA has responded to this demand, representing a significant advantage over national surveys, which do not permit this level of territorial data disaggregation.

By improving the quality of information on the living conditions and health of urban populations, together with the development of diverse strategies for disseminating results, health surveys have contributed to a better understanding of access to and quality of health services and have supported improvements in SUS^21^.

Monitoring patterns of social inequalities in health and estimating their magnitude represent an additional contribution to the planning and management of health actions, enabling the prioritization of the most vulnerable groups in order to reduce health inequities and promote social justice^12^. It is noteworthy that Brazil ranks among the most unequal countries in the world, with a marked concentration of income that was further exacerbated by the COVID-19 pandemic, resulting in a deterioration of living and health conditions among the most vulnerable population segments^22^. This situation also affects MSP, which, despite being the largest city in the country and one of the main economic centers of Latin America, exhibits alarming levels of social inequality^23^. One striking aspect is the disparity in average age at death, reaching a difference of up to 23 years between districts located in the most affluent areas of the city (Jardim Paulista), and those in the far northwest (Anhanguera). These disparities are also reflected in several other indicators, including the utilization of health services, with longer waiting times for tests and medical consultations^24^. Such data are essential for understanding the specific needs of each region and for informing the development of public policies aimed at reducing these inequalities.

The main objectives of ISA Capital-SP 2024 were to assess health status and the use of health services, as well as to identify social inequalities and priority issues, thereby contributing to the improvement of public policies according to health regions.

Considering the challenges associated with maintaining health surveys, the objectives of this study are to describe the methodological procedures adopted for the development of ISA Capital-SP 2024, from planning and sampling to the operational aspects required to enable fieldwork, as well as to report experiences and challenges encountered.

## METHODS

ISA is a cross-sectional study conducted through a population-based household health survey, employing a complex probabilistic sample representative of the urban population of MSP.

ISA 2024, in its fourth edition, was made possible through the continuation of the partnership established in previous editions between the São Paulo Municipal Health Secretariat (*Secretaria Municipal da Saúde de São Paulo –* SMS-SP) and a group of academic institutions, formalized through an agreement with the School of Public Health of Univesidade de São Paulo (*Faculdade de Saúde Pública da Universidade de São Paulo* – FSP-USP). This collaborative model has enabled the pooling of financial and technical resources, involving as key actors professionals from the Epidemiology and Information Coordination (*Coordenação de Epidemiologia e Informação –* CEInfo) of SMS-SP, as well as a group of researchers and other health professionals with experience in conducting this survey, in addition to the university's infrastructure made available to support this initiative.

### Study participants

The reference population consisted of individuals aged 10 years old or older, not institutionalized, and residing in permanent private households located in census tracts within the high-density urban area of MSP. Individuals who did not speak Portuguese were excluded.

### Sampling plan

A two-stage stratified probability sampling design was employed, using cluster sampling with census tracts as the primary sampling units and households as the secondary sampling units.

The strata were defined based on the six Regional Health Coordination Offices (*Coordenadorias Regionais de Saúde –* CRS) of MSP: Central, East, North, West, Southeast, and South, which constituted the study domains. For sample planning purposes, additional domains were established according to age and gender groups: adolescents (10 to 19 years), adult males and females (20 to 59 years), and older adults (60 years old or older). In total, 24 domains were defined, encompassing both geographic and demographic domains.

For operational purposes, the total sample size was set at 5,000 individuals. To ensure that each CRS had comparable analytical capacity, it was determined that samples from each region should reach the largest feasible size (approximately 830 individuals). Initially, the sample allocation was proportional to the population of each age and gender domain within each region. Ho ever, to allow for more precise estimates in the "adolescent" and "older adult" domains, their representation in the sample was increased, while the proportion of adult males and females was reduced, resulting in a revised sample distribution (Table 1).

**Table 1:** Planned sample size for the ISA-Capital SP 2024 health survey, according to domains of interest, Municipality of São Paulo.

CRS	Interviews				
	10 to 19	20 to 59	20 to 59	60 +	Total
		male	Female		
Center	160	213	203	240	816
East	247	216	213	161	837
North	222	209	209	196	836
West	182	199	201	251	834
Southeast	193	203	202	237	835
South	244	216	215	160	835
**Total**	**1,248**	**1,255**	**1,244**	**1,246**	**4,993**

CRS: *Coordenadorias Regionais de Saúde.*

A minimum of 150 interviews was established to estimate a proportion of 0.50, with a sampling error of 0.10, considering a 95% confidence level and a design effect of 1.5. This calculation was based on the algebraic expression used to determine the minimum sample size for estimating proportions in complex samples (Cochran, pp. 50 and 85): $n=\frac{P\cdot \left(1-P\right)}{{\left(d/z\right)}^{2}}\cdot deff$ , where *n* is the sample size, *P* is the parameter to be estimated, *z* = 1.96 corresponds to the value of the standard normal distribution for a 95% confidence level, *d* is the sampling error, and *deff* is the design effect^25^. The design effect was estimated based on results observed in a previous survey conducted in MSP.

In the first stage, 30 census tracts were randomly selected in each CRS, with probability proportional to size, measured by the number of permanent private households recorded in the 2022 Demographic Census^26^. A total of 33 tracts were initially selected in each CRS, of which 3 were retained as a reserve in case sample supplementation was required. By the end of the fieldwork, 187 tracts had been used.

In the second stage, households were selected by systematic random sampling based on enumeration, that is, the list of households compiled during fieldwork. No intra-household random sampling was performed; all individuals belonging to the domain for which the household was selected were included. The absence of intra-household random sampling has been adopted in all editions of the ISA, as this design is considered superior in terms of efficiency and accuracy^27^. The addresses visited for questionnaire administration were recorded in tablets.

A subsample was also selected for the application of a 24-hour Dietary Recall (24hDR) to assess food consumption on the previous day, with a sample size of 300 individuals in each domain. One in four listed addresses was included in the sample for the 24hDR application. The sampling method used was systematic, with a random starting point between 1 and 4.

### Data collection instruments

The following data collection instruments were used:

1. Maps generated in Google Maps and spreadsheets printed for the listing of municipalities and selected census tracts, constructed according to data provided by the Brazilian Institute of Geography and Statistics (*Instituto Brasileiro de Geografia e Estatística* – IBGE) (census tract mesh^28^ and address database)^29^ for the year 2021.
2. Monitoring spreadsheets regarding the status of the selected households, including the frequency of visits carried out and the reasons for failure to identify the eligible population or to conduct interviews.
3. A standardized electronic questionnaire comprising:
  3.1. Sections of questions related to household and family characteristics, such as sociodemographic aspects, food insecurity, and the presence of animals. This section was preferably answered by the resident identified as the household head.
  3.2. Sections of questions for all selected household members, including information on self-reported morbidity in the previous two weeks, use of health services, medication use, mental health, chronic diseases, physical disabilities, cancer screening tests, accidents, violence, and health-related behaviors, among others. These questions were answered directly by the selected resident.
4. A 24hDR form and printed images of portion sizes. These questions were answered by the selected resident in the subsample. The data were recorded by interviewers following the Multiple Pass Method^30^.

For the current survey, meetings were held to review the questionnaires previously used in the ISA, involving, in addition to the researchers directly responsible for the study, specialists and technicians from various departments of SMS-SP, particularly CEInfo. Overall, compatibility with previous ISA versions was maintained, while contemporary topics were incorporated, including changes related to morbidity and the use of health services resulting from the COVID-19 pandemic, food insecurity, and newly relevant immunizations (Chart 1).

**Chart 1:** Description of the themes/modules of the household questionnaires of the ISA-Capital health survey. Municipality of São Paulo, 2003, 2008, 2015, and 2024.

Themes		ISA-Capital			
		2003	2008	2015	2024
**Self-reported morbidity and disabilities**		x	x	x	x
	Morbidity in the past 2 weeks	x	x	x	x
	Chronic diseases	x	x	x	x
	Health problems: complaints and symptoms	x	x	x	x
	Disabilities	x	x	x	x
	COVID-19				x
**Accidents and violence**		x		x	x
	Traffic accidents	x		x	x
	Falls	x		x	x
	Other types of accidents	x		x	x
	Violence	x		x	x
**Emotional health**		x	x	x	x
**Health and well-being**				x	x
**Use of health services**		x	x	x	x
	Use of health services	x	x	x	x
	Hospitalizations and surgeries	x	x	x	x
	Health insurance plans	x	x	x	x
	Oral health	x		x	x
**Preventive exams**		x	x	x	x
	Cervical cancer – Pap smear			x	x
	Breast cancer – mammography			x	x
	Prostate cancer – PSA and digital rectal exam			x	x
	Colorectal cancer – fecal occult blood test and colonoscopy			x	x
**Immunization**				x	x
	Hepatitis B			x	x
	Measles, mumps, and rubella			x	x
	Yellow fever				x
	Adult double vaccine				x
	COVID-19				x
	Influenza (flu)			x	x
	HPV			x	x
**Medication use**		x		x	x
**Health-related behaviors**		x	x	x	x
	Diet		x	x	x
	Physical activity	x	x	x	x
	Smoking	x	x	x	x
	Alcohol consumption	x	x	x	x
**Socioeconomic characteristics of the respondent**		x	x	x	x
**Family and household characteristics**		x	x	x	x
**Socioeconomic characteristics of the household head**		x	x	x	x
	Food insecurity				x
	Vaccination of children (0-6 years of age)	x			
	Vaccine hesitancy in children				x
**Presence of animals**		x	x	x	x
**Health expenditures**		x	x	x	
**Maternal and child health**		x	x		
**Family health program**		x			
***Dengue***		x			

Furthermore, the questionnaire and its administration were pretested, and the necessary adjustments were made. As this was the fourth edition of the ISA, conducted with the same field coordination team with accumulated experience in the study, it was determined that a pilot study was not necessary.

### Data collection procedures

Data collection was conducted using an electronic questionnaire developed on the Research Electronic Data Capture (REDCap) platform, version 14.6.1^31^, and administered by a team of interviewers, supervisors, and a field coordinator, predominantly holding higher education degrees.

Interviewers and supervisors received centralized, in-person training, comprising both theoretical and practical components, to standardize instrument administration and prepare for fieldwork. The training was delivered by the research team, field coordinators, the professional responsible for REDCap, and technicians from SMS-SP. Interviews were conducted face-to-face using tablets, primarily in participants’ homes and, to a lesser extent, via video calls. The latter option was introduced on an experimental basis to reduce refusals in high socioeconomic areas.

Upon arrival at the selected household, the interviewer explained the objectives and importance of the study, completed a form collecting information such as the number of residents, gender, age, and relationship, and verified whether any resident met the age and gender criteria for which the household had been selected. If no residents were present, the interview was terminated without administering any questions.

Interviews were scheduled on a day and time according to the convenience of each eligible resident. In cases where residents could not be located, at least four visits to each household were planned (at different days and times), before the attempt was discontinued. To minimize non-response, several strategies were implemented: (a) legitimization (identification of interviewers and the possibility of verifying their credentials on the study website); (b) communication (distribution of letters to residents and building managers and the dissemination of information through various media outlets); and (c) persistence (rotation of interviewers in areas with greater access difficulties, weekend fieldwork, and standby teams assigned to hard-to-reach locations and areas with higher refusal rates).

Interviews were conducted between August 2023 and December 2024. The obtained sample and its distribution according to the study domains are presented in Table 2.

**Table 2:** Obtained sample size for the ISA-Capital SP 2024 health survey, according to study domains, Municipality of São Paulo.

CRS	Interviews				
	10 to 19	20 to 59	20 to 59	60 +	Total
		male	female		
Center	132	200	215	238	785
East	294	215	231	189	929
North	222	164	239	259	884
West	198	164	183	225	768
Southeast	251	223	254	358	1,086
South	300	224	273	223	1,020
**Total**	**1,397**	**1,190**	**1,395**	**1,492**	**5,472**

CRS: *Coordenadorias Regionais de Saúde.*

### Data monitoring and quality control

The questionnaires completed on the tablets were transmitted daily to the REDCap platform installed on the FSP-USP server. The use of this platform enabled online access to and monitoring of the database, allowing the tracking of interview progress, refusals, and postponements.

Data quality control was conducted with each questionnaire submission, which was subsequently verified by the field supervision team. Concurrently, the database underwent routine consistency checks and review. For data validation, 5% of the sample was randomly selected for telephone verification.

The 24DR data underwent a review process conducted by the nutrition team to identify potential data entry errors and to convert household measures of foods and beverages into units of weight (g) or volume (mL). Subsequently, the data were entered into a specific software program — Nutrition Data System for Research, version 2021 — to estimate energy and nutrient intake and to quantify the foods and ingredients consumed. To identify possible errors in data collection and processing, a consistency analysis of the dietary data was performed in accordance with the manual for assessing food consumption in population studies^32^. A standardized data entry protocol was applied to all reported foods, eliminating the need for duplicate data entry in the software.

### Data analysis

Data analysis was performed using statistical software with modules for complex sampling, allowing the incorporation of design features such as stratification, selection of primary sampling units, and weighting. For the purposes of statistical inference, each individual in the sample was assigned a weight composed of the following components: 1. Design weight, which accounts for the sampling fractions of the two selection stages (census tract and household), whereby the weight of each respondent can be interpreted as the number of individuals in the population represented by that respondent^33^; 2. adjustment for non-response, incorporating the response rates observed in the sectors; and 3. Post-stratification adjustment, based on the distribution of the sample by gender, age group, and CRS, in accordance with the population distribution of MSP in the 2022 Demographic Census.

To analyze variables related to the household population, such as the presence of food insecurity and the number of dogs and cats, a household-level database was created. The design weights, calculated based on the sampling fractions of the selection process, were adjusted for non-response rates in the census tracts and calibrated to the total number of households per CRS, according to the 2022 Census.

For the estimation of means and proportions, it is recommended to exclude estimates with a coefficient of variation greater than 30%, as these indicate low statistical precision.

### Ethical considerations and dissemination of results

The project was approved by the Research Ethics Committees of FSP-USP (CAAE 67818623.6.0000.5421) on April 11, 2023, and of SMS-SP (CAAE 67818623.6.3001.0086) on June 20, 2023. Prior to each interview, informed consent was obtained, with participants providing their signature after reading the Informed Consent Form. For individuals under 18 years of age, an Assent Form was administered.

For the analysis phase, all data will be anonymized and analyzed in aggregate form.

The results will be made available to the general public and policymakers through institutional dissemination via the communication channels of SMS-SP and FSP-USP, as well as through various media formats, including seminars, thematic bulletins, podcasts, and the publication of scientific articles in national and international journals. These strategies aim to ensure transparency, promote social legitimacy, and support the planning of health actions. The dissemination of results represents an ethical and strategic component of the ISA, aligned with the principles of Public Health and SUS.

#### Data availability statement:

The entire dataset supporting the results of this study has been published in the article itself.

## DISCUSSION

The development of the fourth edition of ISA Capital-SP continues a line of research focused on analyzing trends in health conditions and identifying patterns of health service utilization among the population residing in urban areas of MSP.

The main challenges included maintaining the partnership between the university and the public administration while meeting administrative expectations; adapting to emerging needs, trends, and demands while preserving a concise questionnaire; and sustaining effective communication with the population to reduce refusals and increase trust in the use of data for the development and improvement of public policies.

The recommended five-year interval between editions could not be maintained due to difficulties in securing financial resources and the prioritization of other initiatives. However, the current edition made it possible to complete a data series covering the last 22 years.

Since the 2015 ISA, the decision to adopt a sampling design capable of generating estimates at the regional level (CRS) has been confirmed as appropriate, particularly in light of the need to produce intramunicipal indicators for a city with the size and social and geographic inequalities of MSP. In the present edition, the intramunicipal perspective was expanded from five to six health regions, representing an additional challenge due to the increased sample size.

Difficulties in obtaining interviews in household surveys have increased, requiring the adoption of multiple and diverse strategies to address non-response^13^, such as local communication with both health services and the population.

The implementation of the ISA Capital-SP research series has contributed to the expansion of researcher training, providing postgraduate opportunities for health professionals engaged in knowledge production based on ISA data. In parallel, and in alignment with the objectives of funding programs related to public policies, it has also enabled the strengthening of training for health managers and workers in data analysis and the generation of evidence to support decision-making. However, it is important to recognize that this process remains challenging, particularly with regard to the dissemination and appropriation of results by managers as inputs for the planning and evaluation of public health policies.

It is noteworthy that the very constitution of the research group, originating from diverse segments and characterized by strong cohesion and collaboration, together with the involvement of professionals from technical areas and the field of strategic information, has contributed to the quality and maturity of this process, supporting its sustainability, excellence, and longevity.

Jointly conducted activities, such as seminars, workshops, and conceptual alignment meetings, have functioned as spaces for exchange, enabling the avoidance of previously encountered difficulties and fostering greater confidence in the process undertaken. The accumulated experience of this trajectory has contributed to the construction of the research group's identity, which seeks new directions and meanings while valuing and respecting the legacy of prior work. This connection between the experience of professionals involved in previous editions and the current group has constituted the foundation of this process^34^.

The scale and complexity of MSP (Medical Survey of Public Health) require continuous monitoring of the health situation to support both the planning of medium- and long-term actions and the guidance of emergency responses. The recognition of surveys as an important source of information for this purpose has strengthened the link among the key actors involved in this process: management, academia, and the population. Finally, this approach advances the potential to reduce the time between knowledge production and its effective application in the development of public policies capable of transforming reality and, consequently, improving population living conditions.

## References

[1] Malta DC, Leal MC, Costa MFL, Morais OL (2008). Inquéritos Nacionais de Saúde: experiência acumulada e proposta para o inquérito de saúde brasileiro. Rev Bras Epidemiol.

[2] Victora CG (2022). Por que precisamos de inquéritos populacionais sobre saúde? Cad. Saúde Pública.

[3] Viacava F (2002). Informações em saúde: a importância dos inquéritos populacionais. Ciên Saude Colet.

[4] Silva VSTM, Pinto LF (2021). Inquéritos domiciliares nacionais de base populacional em saúde: uma revisão narrativa. Ciên Saude Col.

[5] Instituto Nacional de Estatística (INE) (2016). Inquérito Nacional de Saúde: 2014 [Internet].

[6] Cohen RA, Terlizzi EP, Martinez ME Health Insurance Coverage: Early Release of Estimates from the National Health Interview Survey, 2018 [Internet].

[7] He H, Pan L, Pa L, Cui Z, Ren X, Wang D (2018). BData Resource Profile: The China National Health Survey (CNHS). Int J Epidemiol.

[8] European Commission (2020). Eurostat. European Health Interview Survey (EHIS wave 3). Methodological manual: 2020 edition (re-edition).

[9] NHS (2024). Health Survey for England, 2022 Part 2 [Internet].

[10] Brasil (1990). Lei n° 8.080, de 19 de setembro de 1990. Dispõe sobre as condições para a promoção, proteção e recuperação da saúde, a organização e o funcionamento dos serviços correspondentes e dá outras providências.

[11] Brasil (2014). Ministério da Saúde. Portaria n° 1.271, de 6 de junho de 2014. Define a Lista Nacional de Notificação Compulsória de doenças, agravos e eventos de saúde pública nos serviços de saúde públicos e privados em todo o território nacional, nos termos do anexo, e dá outras providências.

[12] Barros MBA (2008). Inquéritos domiciliares de saúde: potencialidades e desafios. Rev Bras Epidemiol.

[13] Viacava F, Dachs N, Travassos C (2006). Os inquéritos domiciliares e o Sistema Nacional de Informações em Saúde. Ciênc Saúde Coletiva.

[14] Stopa SR, Szwarcwald CL, de Oliveira MM, Gouvea ECDP, Vieira MLFP, de Freitas MPS (2020). BPesquisa Nacional de Saúde 2019: histórico, métodos e perspectivas. Epidemiol Serv Saude.

[15] Vargas AMD, Teixeira DSC, Alves MCGP, Alencar GP, Bernal RTI, Vasconcelos M (2025). BMethodological aspects of national surveys in Brazil: contributions to the debate on oral health surveillance. Braz Oral Res.

[16] de Oliveira MM, Campos MO, de Andreazzi MAR, Malta DC (2017). Características da Pesquisa Nacional de Saúde do Escolar – PeNSE. Epidemiol Serv Saude.

[17] Carvalheiro JR (1975). Levantamento de condições de saúde por entrevistas domiciliárias [tese de livre-docência].

[18] Carandina L, Sanches O, Carvalheiro JR (1986). Análise das condições de saúde e de vida da população urbana de Botucatu, SP: I - Descrição do plano amostral e avaliação da amostra. Rev Saúde Pública.

[19] Cesar CLG, Tanaka OY (1996). Inquérito domiciliar como instrumento de avaliação de serviços de saúde: um estudo de caso na região sudoeste da área metropolitana de São Paulo, 1989-1990. Cad Saúde Pública.

[20] Cesar CLG, Carandina L, Alves MCGP, Barros MBA, Goldbaum M (2005). Saúde e condição de vida em São Paulo. Inquérito multicêntrico de saúde no Estado de São Paulo- ISA-SP.

[21] Carvalho MS, Mello AC, Rabello RS, Lima CRA (2016). Inquérito de Saúde na Esfera Local: colocando em prática.

[22] Guzzo RSL, Souza VLT, Ferreira ÁLMCM (2022). A pandemia na vida cotidiana: reflexões sobre os impactos sociais e psicológicos à luz da perspectiva crítica. Estud Psicol.

[23] Rede Nossa São Paulo (2023). Mapa da Desigualdade ganha novo formato e agora traz a classificação dos 96 distritos de São Paulo – Rede Nossa São Paulo [Internet].

[24] Abrahão J, Pantoja I (2024). Desafios da gestão municipal para redução das desigualdades na cidade de São Paulo. Estud Av.

[25] Cochran WG (1977). Sampling techniques.

[26] Instituto Brasileiro de Geografia e Estatística (IBGE) (2019). Diretoria de Pesquisas. Coordenação de Trabalho e Rendimento. Pesquisa Nacional de Saúde 2019. Questionário dos moradores do domicílio.

[27] Alves MCGP, Escuder MML, Claro RM, da Silva NN (2014). Sorteio intradomiciliar em inquéritos de saúde. Rev Saúde Pública.

[28] Instituto Brasileiro de Geografia e Estatística (IBGE) Malha de setores censitários por UF. Censo Demográfico de 2022 [Internet].

[29] Instituto Brasileiro de Geografia e Estatística (IBGE) Base de Faces de Logradouros por UF/Municípios – Censo Demográfico de 2022 [Internet].

[30] Guenther PM, Demaio TJ, Ingwersen LA, Berlin M (1995). The multiple-pass approach for the 24-hour recall in the Continuing Survey of Food Intakes by Individuals (CSFII) 1994-1996.

[31] Harris PA, Taylor R, Thielke R, Payne J, Gonzalez N, Conde JG (2009). Research electronic data capture (REDCap) a metadata-driven methodology and workflow process for providing translational research informatics support. J Biomed Inform.

[32] Fisberg RM, Marchioni DML (2012). Manual de avaliação do consumo alimentar em estudos populacionais: a experiência do inquérito de saúde em São Paulo (ISA).

[33] Alves MCGP, Escuder MML, Goldbaum M, Barros MBA, Fisberg RM, Cesar CLG (2018). Plano de amostragem em inquéritos de saúde, município de São Paulo, 2015. Rev Saude Publica.

[34] Nora P (1993). Projeto História.

