# Supplementary material for: Population-Based Health Survey in the municipality of São Paulo 2024: methodological and operational aspects
Source: Rev Bras Epidemiol. 2026 Jun 15;29:e260022. doi: 10.1590/1980-549720260022 (PMC13267524; doi:10.1590/1980-549720260022)
Supplement: Translation

# Translation: Inquérito de saúde de base populacional no município de São Paulo, 2024: aspectos metodológicos e operacionais

**DOI:** 10.1590/1980-549720260022.2

**Authors:** Chester Luiz Galvão Cesar, Katia Cristina Bassichetto, Zilda Pereira da Silva, Marilia Cristina Prado Louvison, Regina Mara Fisberg, Maria Cecilia Goi Porto Alves, Maria Mercedes Loureiro Escuder, Gizelton Pereira Alencar, Regina Tomie Ivata Bernal, Edige Felipe de Sousa Santos, Olinda do Carmo Luiz, Moises Goldbaum

**Affiliations:** I Universidade de São Paulo, Faculdade de Saúde Pública – São Paulo (SP), Brasil.; II Secretaria de Estado da Saúde de São Paulo, Instituto da Saúde – São Paulo (SP), Brasil.; III Universidade Federal de Minas Gerais, Escola de Enfermagem, Programa de Pós-Graduação – Belo Horizonte (MG), Brasil.; IV Universidade de São Paulo, Faculdade de Medicina – São Paulo (SP), Brasil.

**Keywords:** Inquéritos epidemiológicos, Métodos, Estudos transversais, Doenças crônicas, Uso de serviços de saúde

## Abstract

Este artigo apresenta o histórico e os procedimentos metodológicos adotados para a construção e o desenvolvimento do inquérito domiciliar de saúde de base populacional ISA Capital-SP 2024, desde o seu planejamento, a amostragem e os aspectos operacionais para a viabilização do campo. Os dados foram coletados por amostragem probabilística estratificada, com sorteio de conglomerados em dois estágios: setores censitários e domicílios. Os estratos foram formados pelas seis regiões de saúde do Município de São Paulo (MSP). Foram entrevistadas as pessoas com 10 anos de idade ou mais, residentes em domicílios particulares permanentes, na área urbana do MSP. O questionário foi composto por questões organizadas em 13 blocos temáticos, como: morbidade referida, uso de serviços, exames de detecção precoce de neoplasias, imunização, uso de medicamentos, comportamentos de saúde, presença de animais, entre outros. Os inquéritos de saúde, quando realizados com periodicidade constante, contribuem para estudos de tendência da situação de saúde e para a identificação de diferentes padrões de utilização de serviços de saúde pela população, complementando os dados dos sistemas oficiais de informação. O ISA Capital-SP constitui-se em um importante instrumento de apoio ao monitoramento das condições de saúde, ao planejamento e à formulação de políticas públicas no âmbito municipal e intramunicipal, especialmente considerando a necessidade de gerar indicadores regionais para uma cidade com as desigualdades sociais e geográficas do MSP.

## INTRODUÇÃO

A produção de informações de diversas fontes possibilita expandir o conhecimento sobre a complexidade dos problemas de saúde, permitindo a expressão de indicadores para o monitoramento contínuo. Os inquéritos de saúde ampliam o olhar na análise dos dados dos sistemas de informações e possibilitam medir comportamentos que influenciam hábitos de saúde e exposições a riscos, entender os padrões de busca por serviços assistenciais, medir problemas de saúde que resultam em grandes cargas de morbidade, e estratificar os indicadores por subgrupos populacionais para evidenciar desigualdades em saúde^1,2^.

Nesse sentido, os inquéritos de saúde de base populacional têm ocupado posição de destaque na produção de informação em saúde desde a década de 1960, em diversos países da Europa, nos Estados Unidos e na China, em diferentes contextos, envolvendo centenas de milhares de domicílios e participantes^3–9^.

No Brasil, há uma política em curso que reconhece a necessidade de inquéritos periódicos para gerar informações não disponíveis em outras fontes^10,11^. Desde a década de 1970, diversos inquéritos domiciliares, incluindo os da saúde, têm sido realizados, sendo os principais: Pesquisas de Orçamento Familiar (POF); Pesquisa Nacional sobre Demografia e Saúde (PNDS); Suplementos Saúde da Pesquisa Nacional por Amostra de Domicílios (Pnad); Pesquisa Nacional de Saúde (PNS), que produz dados sobre a situação de saúde e os estilos de vida, bem como sobre o acesso e uso dos serviços, ações preventivas, continuidade dos cuidados e financiamento da assistência; Pesquisa Nacional de Saúde Bucal (SB Brasil); Pesquisa Nacional da Saúde do Escolar (PeNSE), importante iniciativa para compreender a saúde e o bem-estar dos adolescentes^12–16^.

Especialmente no âmbito da saúde, considerando as dimensões do país, os inquéritos têm possibilitado a ampliação do conhecimento sobre a variação das estimativas dos indicadores nacionais e regionais de saúde e o aprofundamento de questões que necessitam de investigações mais específicas. As informações produzidas sobre os determinantes, condicionantes e as necessidades de saúde subsidiam a formulação e o aprimoramento de políticas no Sistema Único de Saúde (SUS)^14^.

O estado de São Paulo tem, também, um histórico de realização de inquéritos de saúde, iniciando em Ribeirão Preto, em 1974, pelo Levantamento Contínuo de Condições de Saúde por Entrevistas Domiciliares (primeiro no âmbito da saúde no Brasil^17^); o Projeto Morbidade, em 1990, na região sudoeste da Grande São Paulo^18,19^; o Inquérito Multicêntrico do Estado de São Paulo, em 2002, em Botucatu, Campinas, Grande São Paulo e em um distrito de saúde de São Paulo^20^. Outras experiências municipais têm se desenvolvido, como o Inquérito de Saúde de Campinas; e o ISA Capital-SP (2003, 2008 e 2015), no município de São Paulo (MSP). Um aspecto a ser evidenciado neste último é a possibilidade de atender às necessidades de análise em nível intramunicipal da gestão. O ISA, desde 2015, tem contemplado essa demanda, trazendo um diferencial importante em relação às pesquisas nacionais, que não permitem essa desagregação territorial dos dados.

Ao melhorar a qualidade da informação sobre as condições de vida e saúde das populações urbanas, acompanhada do desenvolvimento de estratégias diversificadas de disseminação dos resultados, os inquéritos de saúde têm contribuído para compreender melhor o acesso e a qualidade aos serviços de saúde e subsidiar o aprimoramento do SUS^21^.

O monitoramento de padrões de desigualdades sociais em saúde e as estimativas da magnitude dessas desigualdades representam mais uma contribuição para o planejamento e a gestão das ações em saúde, priorizando os grupos mais vulnerabilizados, no intuito de reduzir as iniquidades em saúde e promover justiça social^12^. Vale ressaltar que o Brasil é o nono país mais desigual do mundo, com forte concentração de renda, o que foi agravado pela pandemia de covid-19, levando à piora das condições de vida e de saúde de segmentos populacionais mais vulnerabilizados^22^. Essa conjuntura atinge também o MSP, que, apesar de ser a maior cidade do país e um dos principais centros econômicos da América Latina, apresenta níveis alarmantes de desigualdade social^23^. Um dos aspectos marcantes refere-se à diferença na idade média ao morrer, que chega a 23 anos entre distritos localizados na região mais rica da cidade (Jardim Paulista) e no extremo noroeste (Anhanguera). Tais disparidades se repetem em muitos outros indicadores, inclusive no uso de serviços de saúde, expressos em maior tempo de espera por exames e consultas^24^. Esses dados são importantes para compreender as necessidades específicas de cada região e para a elaboração de políticas públicas que visem reduzir essas desigualdades.

Os principais objetivos do ISA Capital-SP 2024 foram avaliar o estado de saúde e o uso de serviços, e identificar desigualdades sociais e questões prioritárias como contribuição para a melhoria das políticas públicas, segundo as regiões de saúde.

Considerando os desafios da manutenção dos inquéritos de saúde, os objetivos deste estudo são: descrever os procedimentos metodológicos adotados para o desenvolvimento do ISA Capital-SP 2024, desde o planejamento, a amostragem e os aspectos operacionais para a viabilização do trabalho de campo, bem como compartilhar experiências e desafios.

## MÉTODOS

O ISA é um estudo transversal, realizado por meio de inquérito domiciliar de base populacional em saúde, com amostra probabilística complexa, representativa da população urbana do MSP.

O ISA 2024, em sua 4ª edição, se viabilizou pela continuidade da parceria firmada, em edições anteriores, entre a Secretaria Municipal de Saúde de São Paulo (SMS-SP) e um conjunto de instituições acadêmicas, por meio de um convênio com a Faculdade de Saúde Pública da Universidade de São Paulo (FSP-USP). Tal modalidade tem permitido reunir recursos financeiros e técnicos, envolvendo como principais atores profissionais da Coordenação de Epidemiologia e Informação (CEInfo) da SMS-SP e um grupo de pesquisadores e demais profissionais da saúde que desenvolveram experiência na realização deste inquérito – além da infraestrutura da universidade, colocada à disposição desta iniciativa.

### Participantes do estudo

A população de referência foi composta por indivíduos de 10 anos ou mais de idade, não institucionalizados, residentes em domicílios particulares permanentes, localizados em setores censitários da área urbana de alta densidade do MSP. Foram excluídas as pessoas que não dominavam o idioma português.

### Plano amostral

Foi utilizada amostragem probabilística estratificada por sorteio de conglomerados em dois estágios: setores censitários e domicílios.

Os estratos foram formados pelas seis Coordenadorias Regionais de Saúde (CRS) do MSP: Centro, Leste, Norte, Oeste, Sudeste e Sul, que constituíram os domínios do estudo. Para fins de planejamento da amostra, para grupos de idade e sexo também foram considerados os domínios: adolescentes (10 a 19 anos de idade), adultos do sexo masculino e feminino (20 a 59 anos de idade) e idosos (60 anos ou mais de idade). No total, foram definidos 24 domínios, entre geográficos e demográficos.

Considerou-se, por questões de caráter operacional, que o tamanho total da amostra seria de 5 mil pessoas. Para que as CRS tivessem o mesmo potencial de análise dos dados, decidiu-se que as amostras de cada região deveriam atingir o maior tamanho possível (830 indivíduos). Inicialmente, delimitou-se a distribuição das amostras pelos domínios de idade e sexo, proporcional à população de cada um. Entretanto, para que nos domínios "adolescentes" e "idosos" as estimativas pudessem ser feitas com maior precisão, a participação desses grupos na amostra foi alterada. Considerou-se uma população maior desses domínios e outra, de adultos masculinos e femininos, menor, sendo feita uma nova distribuição da amostra (Tabela 1).

**Tabela 1:** Tamanho planejado da amostra para o inquérito de saúde ISA-Capital SP 2024, segundo os domínios de interesse, Município de São Paulo.

CRS	Entrevistas				
	10 a 19	20 a 59	20 a 59	60 e +	Total
		masculino	feminino		
Centro	160	213	203	240	816
Leste	247	216	213	161	837
Norte	222	209	209	196	836
Oeste	182	199	201	251	834
Sudeste	193	203	202	237	835
Sul	244	216	215	160	835
**Total**	**1.248**	**1.255**	**1.244**	**1.246**	**4.993**

CRS: Coordenadorias Regionais de Saúde.

Determinou-se um número mínimo de 150 entrevistas para estimar uma proporção de 0,50, com erro de amostragem de 0,10, considerando um nível de confiança de 95% e um efeito de delineamento de 1,5. Esse cálculo foi feito com base na expressão algébrica que determina o tamanho mínimo de amostra para a estimação de proporções sob amostras complexas (Cochran, pág. 50 e 85): $n=\frac{P\cdot \left(1-P\right)}{{\left(d/z\right)}^{2}}\cdot deff$ , em que *n* é o tamanho da amostra, *P* é o parâmetro a ser estimado, *z* = 1,96 é o valor na curva normal reduzida referente ao nível de confiança de 95% dos intervalos de confiança, *d* é erro de amostragem, e *deff* é o efeito do delineamento^25^. A estimativa do efeito do delineamento foi feita de acordo com os resultados observados em inquérito anterior realizado no MSP.

No primeiro estágio, foram sorteados 30 setores censitários em cada CRS, com probabilidade proporcional ao tamanho, medido pelo número de domicílios particulares permanentes contabilizados para a realização do Censo Demográfico 2022^26^. Foram sorteados 33 em cada CRS, dos quais 3 ficaram como reserva, caso precisasse complementar a amostra. Ao final do trabalho de campo, foram utilizados 187 setores.

No segundo estágio, os domicílios foram selecionados por sorteio sistemático, com base no arrolamento, ou seja, na listagem de domicílios realizada em campo. Não houve sorteio intradomiciliar; todos os indivíduos pertencentes ao domínio para o qual o domicílio foi sorteado foram incluídos. A ausência de sorteio intradomiciliar tem sido adotada em todas as edições do ISA por se considerar que esses delineamentos são superiores em termos de eficiência e acurácia^27^. Os endereços visitados para aplicação do questionário foram digitados em *tablets*.

Também foi realizada uma subamostra para aplicação de Recordatório Alimentar de 24h (R24h), para medir o consumo alimentar do dia anterior, com tamanho de 300 pessoas em cada domínio. Um a cada quatro endereços arrolados compuseram a amostra para aplicação do R24. O sorteio utilizado foi sistemático com início casual entre 1 e 4.

### Instrumentos de coleta de dados

Foram utilizados os seguintes instrumentos de coleta de dados:

1. Mapas gerados no Google Maps e planilhas impressas para arrolamento dos domicílios dos setores censitários sorteados, construídas de acordo com os dados disponibilizados pelo Instituto Brasileiro de Geografia e Estatística (IBGE) (malha de setores censitários^28^ e base de dados de logradouros)^29^ para o ano de 2021.
2. Planilhas de acompanhamento sobre a situação dos domicílios sorteados, relativa à frequência de visitas realizadas e aos motivos da não identificação da população elegível e da não realização de entrevistas.
3. Questionário padronizado eletrônico englobando:
  3.1. Blocos de questões relativas às características da família e do domicílio, como aspectos sociodemográficos, insegurança alimentar e presença de animais. Esta parte foi respondida preferencialmente pelo morador identificado como responsável pelo domicílio.
  3.2. Blocos de questões para todos os moradores selecionados do domicílio: informações como morbidade referida nas duas últimas semanas, utilização de serviços de saúde, uso de medicamentos, saúde mental, doenças crônicas, deficiências físicas, exames de detecção precoce de neoplasias, acidentes, violências e comportamentos relacionados à saúde, entre outras. Essas questões foram respondidas diretamente pelo próprio morador selecionado.
4. Formulário R24h e imagens dos tamanhos das porções impressas. As questões foram respondidas pelo morador selecionado na subamostra. Os dados foram registrados pelos entrevistadores, seguindo o Método de Passagem Múltipla^30^.

Para o atual inquérito, foram realizadas reuniões para revisão dos questionários anteriormente utilizados no ISA, envolvendo, além dos pesquisadores diretamente responsáveis pela pesquisa, especialistas e técnicos de diversas áreas da SMS-SP, especialmente da CEInfo. No geral, foi mantida a compatibilidade com as versões anteriores do ISA e incluídos temas atuais, como transformações decorrentes da pandemia de covid-19 em relação à morbidade e ao uso de serviços de saúde, à insegurança alimentar, além de novos imunizantes de interesse (Quadro 1).

**Quadro 1:** Descrição dos temas/módulos dos questionários domiciliares do inquérito de saúde ISA-Capital. Município de São Paulo, 2003, 2008, 2015 e 2024.

Temas		ISA-Capital			
		2003	2008	2015	2024
**Morbidade referida e deficiências**		x	x	x	x
	Morbidade em 2 semanas	x	x	x	x
	Doenças crônicas	x	x	x	x
	Problemas de saúde: queixas e sintomas	x	x	x	x
	Deficiências	x	x	x	x
	Covid-19				x
**Acidentes e violência**		x		x	x
	Acidentes de trânsito	x		x	x
	Quedas	x		x	x
	Outros tipos de acidentes	x		x	x
	Violências	x		x	x
**Saúde emocional**		x	x	x	x
**Saúde e bem-estar**				x	x
**Uso de serviços de saúde**		x	x	x	x
	Uso de serviços de saúde	x	x	x	x
	Hospitalizações e cirurgias	x	x	x	x
	Planos de saúde	x	x	x	x
	Saúde bucal	x		x	x
**Exames preventivos**		x	x	x	x
	Câncer de colo de útero – papanicolau			x	x
	Câncer de mama – mamografia			x	x
	Câncer de próstata – PSA e toque retal			x	x
	Câncer colorretal – PSO e colonoscopia			x	x
**Imunização**				x	x
	Hepatite B			x	x
	Sarampo, caxumba e rubéola			x	x
	Febre amarela				x
	Dupla adulta				x
	Covid-19				x
	Gripe/Influenza			x	x
	HPV			x	x
**Uso de medicamentos**		x		x	x
**Comportamentos relacionados à saúde**		x	x	x	x
	Alimentação		x	x	x
	Atividade física	x	x	x	x
	Tabagismo	x	x	x	x
	Consumo de bebidas	x	x	x	x
	**Características socioeconômicas do entrevistado**	x	x	x	x
	**Características da família e do domicílio**	x	x	x	x
	**Características socioeconômicas do responsável pela família**	x	x	x	x
	Segurança alimentar				x
	Vacinação de crianças (0-6 anos de idade)	x			
	Hesitação vacinal de crianças				x
**Presença de animais**		x	x	x	x
**Gastos com saúde**		x	x	x	
**Materno-infantil**		x	x		
**Programa de saúde da família**		x			
**Dengue**		x			

Foram realizados, ainda, o pré-teste do questionário e do aplicativo e os ajustes necessários. Como se trata da quarta edição do ISA, realizada com a mesma equipe de coordenação de campo, com experiência acumulada nessa pesquisa, avaliou-se que não era necessário realizar um estudo-piloto.

### Procedimentos de coleta de dados

A coleta de dados ocorreu por questionário eletrônico, elaborado na plataforma *Research Electronic Data Capture* (*REDCap*) versão 14.6.1^31^ e aplicado por uma equipe de entrevistadores, supervisores e coordenador de campo, com formação predominantemente superior.

Foram realizados treinamentos dos entrevistadores e supervisores, com dimensões teórica e prática, de forma centralizada e presencial, para padronizar a aplicação dos instrumentos e preparar a execução dos trabalhos de campo. O treinamento foi aplicado pela equipe de pesquisadores, coordenação de campo, profissional responsável pelo *REDCap* e técnicos da SMS-SP. As entrevistas foram realizadas com o uso de *tablets*, face a face – na maioria dos casos, no domicílio e, numa pequena proporção, via chamada de vídeo. Essa opção foi introduzida experimentalmente, para minimizar as recusas em regiões de alto nível socioeconômico.

Ao chegar ao domicílio sorteado, o entrevistador explicava os objetivos e a importância da pesquisa, preenchia um quadro com dados como número de moradores residentes, sexo, idade e grau de parentesco; e verificava se algum morador atendia aos critérios do grupo etário e sexo para os quais o domicílio havia sido sorteado. Caso não houvesse morador no domínio, a entrevista era encerrada e não se aplicava nenhuma questão.

As entrevistas foram agendadas em dia e horário conforme a conveniência de cada morador elegível. No caso de não encontrar os moradores, previu-se a realização de quatro visitas a cada domicílio (em dias e horários distintos), no mínimo, antes de desistir.

Para minimizar a não resposta, utilizamos algumas estratégias: (a) legitimação (entrevistadores identificados, com possibilidade de confirmar a identidade no *site*); (b) comunicação (distribuição de cartas a moradores e síndicos de prédios, veiculação de reportagens em diversas mídias) e (c) persistência (rodízio de entrevistadores em setores com maior dificuldade de abordagem, mutirão de final de semana, e plantão nos locais de difícil acesso e de maior recusa).

As entrevistas ocorreram entre agosto de 2023 e dezembro de 2024. A amostra obtida e sua distribuição, segundo os domínios de estudo, constam na Tabela 2.

**Tabela 2:** Tamanho de amostra obtida para o inquérito de saúde ISA-Capital SP 2024, segundo os domínios de estudo, Município de São Paulo.

CRS	Entrevistas				
	10 a 19	20 a 59	20 a 59	60 e +	Total
		masculino	feminino		
Centro	132	200	215	238	785
Leste	294	215	231	189	929
Norte	222	164	239	259	884
Oeste	198	164	183	225	768
Sudeste	251	223	254	358	1.086
Sul	300	224	273	223	1.020
**Total**	**1.397**	**1.190**	**1.395**	**1.492**	**5.472**

CRS: Coordenadorias Regionais de Saúde.

### Monitoramento e controle de qualidade dos dados

Os questionários preenchidos nos *tablets* eram enviados diariamente para a plataforma *REDCap* instalada no servidor da FSP-USP. O uso dessa plataforma permitiu o acesso online e o monitoramento do banco de dados para acompanhar a evolução das entrevistas realizadas, das recusas e dos adiamentos.

O controle de qualidade dos dados foi realizado a cada envio dos questionários – que, por sua vez, foram verificados pela equipe de supervisão de campo. Concomitantemente, o banco de dados passou por um processo rotineiro de consistência e de crítica. Para validação dos dados, realizou-se um sorteio de 5% da amostra para checagem por telefone.

Os dados do R24h passaram por um processo de crítica pela equipe de nutrição para identificar eventuais falhas no preenchimento e converter as medidas caseiras de alimentos e bebidas em unidades de peso (g) ou volume (ml). Na sequência, foram digitados em um programa específico – *Nutrition Data System for Research*, v. 2021, para o cálculo da ingestão de energia e nutrientes, bem como a quantificação de alimentos e ingredientes consumidos. A fim de identificar possíveis erros na coleta e no processamento de dados, foi realizada análise de consistência dos dados dietéticos, conforme o manual de avaliação do consumo alimentar em estudos populacionais^32^. Um padrão de digitação foi utilizado para inserção de todos os alimentos consumidos, não sendo necessária a estratégia de dupla digitação no programa.

### Análise dos dados

A análise dos dados realizou-se em *softwares* de análise estatística que possuem módulos de amostragem complexa e permitem a inclusão de aspectos relacionados ao delineamento: estratificação, sorteio de unidades primárias de amostragem e ponderação.

Para fins de inferência estatística, cada indivíduo da amostra foi associado a um peso, composto pelos seguintes componentes: 1. Peso de delineamento, que leva em conta as frações de amostragem das duas etapas de sorteio (de setor censitário e de domicílio), em que o peso de cada pessoa entrevistada pode ser interpretado como o número de pessoas da população que ela representa^33^; 2. Ajuste pela não resposta, que contempla as taxas de resposta observadas nos setores; 3. Ajuste por pós-estratificação, considerando a distribuição da amostra por sexo, faixa etária e CRS, de acordo com a distribuição da população do MSP no Censo Demográfico 2022.

Para a análise de questões referentes ao conjunto de moradores das unidades domiciliares, como a presença de insegurança alimentar na família e a quantidade de cães e gatos, foi construído um banco de domicílios. Os pesos do delineamento, calculados com base na fração de amostragem do sorteio, foram ajustados pelos percentuais de não resposta nos setores censitários e pelo total de domicílios por CRS, segundo o Censo 2022.

Para obtenção de estimativas de médias e proporções, recomenda-se não considerar válidas medidas cujo coeficiente de variação seja superior a 30%, o que indica baixa precisão estatística.

### Aspectos éticos e divulgação dos resultados

O projeto foi aprovado pelos Comitês de Ética em Pesquisa da FSP-USP (CAAE 67818623.6.0000.5421), em 11/04/2023, e da SMS-SP (CAEE 67818623.6.3001.0086), em 20/06/2023. Antes da realização de cada entrevista, foram solicitadas a concordância e assinatura após a leitura do Termo de Consentimento Livre e Esclarecido. Para menores de 18 anos foi aplicado o Termo de Assentimento.

Para a etapa de análise, todos os dados serão anonimizados e tratados de forma agregada.

Os resultados se tornarão disponíveis para a população em geral e para formuladores de políticas públicas, por meio de divulgação institucional nos canais da SMS-SP e da FSP-USP e em diversas mídias, como seminários, produção de boletins temáticos, *podcasts*, além de publicação de artigos científicos em periódicos nacionais e internacionais. Essas estratégias visam assegurar transparência, legitimidade social e apoio ao planejamento de ações em saúde. A devolutiva dos resultados constitui uma etapa ética e estratégica do ISA, alinhada aos princípios da Saúde Coletiva e do SUS.

#### Declaração de disponibilidade de dados:

Todo o conjunto de dados que dá suporte aos resultados deste estudo foi publicado no próprio artigo.

## DISCUSSÃO

O desenvolvimento da quarta edição do ISA Capital-SP dá continuidade à linha de pesquisa que abrange estudos de tendência da situação de saúde e identifica diferentes padrões de utilização de serviços de saúde pela população residente em áreas urbanas do MSP.

Os principais desafios vivenciados foram: manter a parceria entre a universidade e a gestão pública e atender às expectativas da gestão; ajustar necessidades, novas tendências e demandas, além de manter um questionário sintético; manter comunicação com a população para reduzir recusas, ampliando sua confiança na utilização dos dados para produção e aprimoramento de políticas públicas.

A periodicidade recomendada de cinco anos entre cada edição não pôde ser cumprida, por dificuldade na obtenção de recursos financeiros ou pela priorização de outras iniciativas. Contudo, com a atual edição, foi possível concluir um painel seriado dos últimos 22 anos.

Desde o ISA 2015, verificou-se que a decisão de introduzir um desenho amostral para gerar estimativas para o nível regional (CRS) foi acertada, especialmente considerando a necessidade de gerar indicadores intramunicipais para uma cidade com as dimensões e as desigualdades sociais e geográficas do MSP. Na presente edição, o olhar intramunicipal foi expandido de cinco para seis regiões de saúde, o que representou um desafio adicional, devido ao aumento do tamanho de amostra.

A dificuldade em obter entrevistas tem sido crescente em inquéritos domiciliares, exigindo a adoção de inúmeras estratégias diversificadas para reverter a não resposta^13^, como comunicação local tanto com os serviços de saúde quanto com a população.

A realização da série de pesquisa ISA Capital-SP tem permitido ampliar a formação de pesquisadores, oferecendo oportunidades de pós-graduação a profissionais de saúde que se dedicam à produção de conhecimento, com base nos dados do ISA. Ao lado disso, coerente com os objetivos dos programas de fomento relacionados às políticas públicas, tem sido possível ampliar a formação de gestores e trabalhadores de saúde na análise de dados e na produção de evidências para a tomada de decisão. É importante considerar, entretanto, que esse movimento é bastante desafiador, particularmente do ponto de vista da divulgação e da apropriação dos resultados pelos gestores como subsídios para planejar e avaliar as políticas públicas na área da saúde.

Vale destacar que a própria constituição do grupo de pesquisadores, oriundos de diversos segmentos, com fortes elementos de aglutinação e colaboração, somado ao envolvimento dos profissionais das áreas técnicas e da área de informação estratégica, têm conferido qualidade e maturidade a esse processo e permitido sua sustentabilidade, excelência e longevidade.

As atividades conduzidas em conjunto, como seminários, oficinas e reuniões de alinhamento conceitual, têm se caracterizado como espaços de compartilhamento, permitindo evitar dificuldades vivenciadas anteriormente e obter mais confiança no percurso transcorrido. A memória desse percurso tem contribuído para a construção da identidade deste grupo de pesquisa, que busca novos caminhos e significados, valorizando e respeitando as heranças do trabalho dos que nos antecederam. Essa ligação entre a experiência vivenciada pelos profissionais envolvidos nas edições anteriores e o grupo atual tem sido a base dessa construção^34^.

A dimensão e a complexidade do MSP requerem um monitoramento da situação de saúde que possibilite tanto o planejamento de ações a médio e longo prazo quanto a orientação de ações emergenciais. O reconhecimento dos inquéritos como importante fonte de informações para esse fim tem consolidado a ponte estabelecida entre os elementos envolvidos nesse processo: gestão, academia e população. Por fim, avança na possibilidade de reduzir o tempo entre a produção de conhecimento e o uso efetivo para a produção de políticas públicas que transformem a realidade e, por conseguinte, melhorem as condições de vida da população.
